# The Impact of Mood and Subjective Intoxication on Hangover Severity

**DOI:** 10.3390/jcm9082462

**Published:** 2020-08-01

**Authors:** Joris C. Verster, Lizanne Arnoldy, Aurora J.A.E. van de Loo, Sarah Benson, Andrew Scholey, Ann-Kathrin Stock

**Affiliations:** 1 Division of Pharmacology, Utrecht Institute for Pharmaceutical Sciences (UIPS), Utrecht University, 3584CG Utrecht, The Netherlands; J.C.Verster@uu.nl (J.C.V.); larnoldy@swin.edu.au (L.A.); a.j.a.e.vandeloo@uu.nl (A.J.A.E.v.d.L.); 2 Institute for Risk Assessment Sciences (IRAS), Utrecht University, 3584CM Utrecht, The Netherlands; 3 Centre for Human Psychopharmacology, Swinburne University, Melbourne 3122, Australia; sarahmichellebenson@gmail.com (S.B.); andrew@scholeylab.com (A.S.); 4 Cognitive Neurophysiology, Department of Child and Adolescent Psychiatry, Faculty of Medicine, TU Dresden, Fetscherstr. 74, 01307 Dresden, Germany; 5 Biopsychology, Department of Psychology, School of Science, TU Dresden, Zellescher Weg 19, 01069 Dresden, Germany

**Keywords:** alcohol, hangover, mood, subjective intoxication, stress, neuroticism

## Abstract

The aim of this study was to investigate whether baseline mood and/or mood while drinking have an impact on alcohol hangover severity. A survey was held among *N* = 331 young adults (mean age = 23.6 years, range = 18–35 years). Demographics, alcohol consumption, subjective intoxication, and hangover severity were assessed for the past three days. In addition, mood (baseline, while drinking, and during hangover) was also assessed. *N* = 143 participants reported to be hungover on the day of assessment, *N* = 122 participants reported to have been hungover the previous day (‘yesterday’), and *N* = 87 participants reported to have been hungover two days before the assessment (‘2 days ago’). The analyses revealed that baseline mood and mood while drinking had no relevant effect on the amount of consumed alcohol and did not significantly contribute to hangover severity. However, hangover severity was associated with significantly increased negative affect, particularly with higher levels of subjective stress on the day of the hangover.

## 1. Introduction

Negative baseline mood and mood while drinking have been reported to influence alcohol consumption. This is reflected by reports that situational factors (experiencing negative life events and stress) and intrinsic factors (e.g., neuroticism) can be associated with negative mood [1,2]. Subsequently, certain individuals are more likely to develop negative coping styles, including increased alcohol use [3]. Despite their conjoint effect on mood, it is important to note that trait-like, rather stable, baseline mood may be different from the current affective state while drinking (i.e., state mood) [4], so both should be considered when investigating associations between mood and drinking behavior. Additionally, sex may play a role as women are usually more likely than men to increase their drinking behavior in response to negative affect and stress and to report greater stress relief by drinking [5], but they also suffer more negative affective (long-term) consequences of excessive alcohol consumption [6,7]. Finally, it should be kept in mind that while alcohol intake may be motivated by the wish for relief of negative affect, it may also enhance trait or state mood and emotions, thus constituting a bidirectional association [8].

The functional link between stress and alcohol consumption has repeatedly been demonstrated via changes in the hypothalamic–pituitary-adrenal (HPA) axis, especially with respect to the corticotropin-releasing factor (CRF) [6,9]. Specifically, it has been found that higher levels of stress as well as reduced sensitivity of the HPA axis escalate alcohol consumption and increase the likelihood of binge drinking [10]. Lastly, a genetic study conducted in *Drosophila* has suggested that alcohol hangover/tolerance development and the response to stress may be partly mediated by the same molecular pathway [11].

Due to the phenomenological (affective) and functional (neurobiochemical/neuroendocrinological) overlap between mood, stress, and hangover, mood and subjective stress may be predictors of hangover severity. Despite the close link between mood and alcohol consumption, only very few studies have investigated the effects of baseline and/or current mood on experiencing hangovers.

Hangover is the most commonly reported negative consequence of alcohol consumption [12] and has been defined as the combination of negative mental and physical symptoms which may be experienced after a single episode of alcohol consumption, starting when blood alcohol concentration (BAC) approaches zero [13,14]. In a sample of male alcohol use disorder patients and social drinkers, Gunn [15] reported that negative attitudes towards drinking alcohol and feeling guilty about drinking were associated with experiencing more severe hangovers, but this association does not allow for any conclusions on causality. More recently, a regression analysis by Piasecki et al. [16] revealed that experiencing depressive symptoms was associated with both current and future hangover susceptibility, and Royle et al. [17] found that drinkers who had higher levels of pain catastrophizing reported experiencing more severe hangovers. However, research in the area of alcohol hangover is still severely limited. Hence, more studies need to be conducted to elucidate to what extent personality aspects and baseline mood have an impact on the susceptibility to the occurrence and severity of hangovers.

While it is commonly reported that mood and emotions are negatively affected during the hangover state [18,19,20], the extent to which mood while drinking impacts hangover severity has largely been neglected in hangover literature. In fact, only two studies investigated the direct impact of mood during drinking on next-day hangover in the same sample [21,22]. The first article [21], which included *N* = 1266 subjects (randomly selected from the Tecumseh Community Health Study), reported positive correlations between hangover symptom frequency and psychosocial factors, including negative life events, neuroticism, guilt about drinking, feeling depressed while drinking, and being angry while drinking. In the second paper, Harburg et al. [22] excluded all sober subjects from their dataset, i.e., subjects who reported ‘never’ being ‘tipsy, high, or drunk’. When these were removed and the data of the remaining *N* = 1104 subjects were re-analyzed, the correlations between hangover symptom frequency and mood while drinking were less pronounced. Significant correlations were reported for neuroticism, guilt about drinking, drinking to escape, negative life events, and feelings of depression and anger while drinking. A stepwise linear regression analysis including all of the assessed variables was performed separately for men and women. Both analyses yielded a model with only modest predictive validity (19% in men and 21% in women) for the variance in the reported frequency of hangover symptoms. The analyses showed that with regard to the contribution of individual variables to the explained variance, guilt about drinking was the strongest predictor of hangover severity (9% in men and 11% in women), followed by neuroticism (4% in both men and women), being angry when high/drunk (3% in men and 2% in women), and negative life events (2% in men and 1% in women). In men, being depressed when high/drunk (1%) and the amount of consumed ethanol (<1%) further contributed to the model. In women, additional significant predictors were being younger (2%) and having experienced being drunk for the first time at a younger age (1%). Together, these findings suggest that mood during drinking has relatively little impact on experiencing hangover symptoms.

However, there are several issues that complicate the interpretation of the results presented by Harburg et al. [21,22]. For example, while they excluded subjects who reported not being drunk (but could still have had a hangover), 23% of the subjects who reported experiencing no hangover remained in the sample that underwent statistical analyses. As a consequence, the Pearson’s correlations and forward stepwise multiple regression used to examine the data may have produced less pronounced associations in this zero-inflated sample [23]. Moreover, the authors assumed that they measured hangover severity, but the Hangover Sign Index (HSI) actually assesses the frequency with which eight different hangover symptoms (‘headache or hangover’, ‘loss of appetite’, ‘diarrhea’, ‘stomach pains’, ‘anxiety’, ‘blackout or loss of memory’, ‘tremors or hand shaking’, and ‘thoughts of suicide’) occur after drinking, rather than their severity. In the study by Harburg et al. [21,22], subjects indicated whether or not they experienced these symptoms on the day following the latest occasion when they were drunk. The specific combination of experienced hangover symptoms was used to estimate hangover severity. The six levels of hangover severity distinguished by Harburg et al. included (1) ‘no signs’ (gets drunk, but reports no hangover signs), (2) weak (any or all of these three symptoms: headache, diarrhea, and loss of appetite), (3) ‘mild’ (anxiety and/or stomach pains), (4) strong (any one of blackout, tremor, and thoughts of suicide), (5) ‘very strong’ (anxiety plus any one of blackout, tremor, and thoughts of suicide), and (6) ‘severe hangover’ (two or more of blackout, tremor, and suicidal thoughts). The validity of the HSI to reliably measure hangover severity can be questioned in several ways. First, the frequency of symptom occurrence does not tell us anything about their severity. Second, the HSI contains items that are not hangover signs, but signs of intoxication (e.g., blackouts). Third, it omits several core symptoms of the hangover state (e.g., fatigue or nausea), while including other signs such as ‘thoughts of suicide’, which are seldom regarded as hangover symptoms in the scientific literature [19,20]. There is great variability with regard to the presence and severity of hangover symptoms [19,24] and a recent study suggested that composite hangover scales are less accurate as they may over- or under-represent core symptoms and/or hangover-irrelevant symptoms [24]. In addition, the HSI score does not account for the impact of the experienced symptoms.

Taken together, it is important to replicate and improve the study by Harburg et al. by including a valid assessment of hangover severity to infer whether mood while drinking has an impact on the presence and severity of next-day alcohol hangover. Therefore, the current study aimed to verify and extend the observations by Harburg et al. in an international sample of young adults by applying a 1-item overall hangover severity scale, which is regarded to be superior to composite symptom scores [24]. Specifically, we investigated whether baseline mood, mood while drinking, and mood during hangover were associated with and/or predicting current and retrospective hangover severity.

## 2. Methods

In August 2018, a survey was conducted among an international sample of young adults who came to Fiji either for work or holidays. Both men and women within the age range of 18–35 years were included. The young adults were approached at Wailoaloa Beach and asked to complete a survey.

Subjects who were willing to participate and sufficiently understood the English language completed the survey on location. The location was chosen because a relatively large number of young adults congregated here to spend a holiday or relax after work.

The survey was anonymous and subjects did not receive an incentive for completing the survey. The investigator was present to clarify any issues arising from English not being the participants’ mother tongue. The study was conducted by Utrecht University, informed consent was obtained from all subjects, and the Ethics Committee of the Faculty of Social and Behavioral Sciences of Utrecht University granted ethical approval (approval code FETC17-061).

The survey collected demographic information, including age, gender, height, and weight to compute body mass index (BMI), and usual weekly alcohol intake. The survey contained guidance about standard drinking sizes, and how to convert, for example, a bottle of wine into standardized alcohol units, which contain 10 g of alcohol each.

To assess the past year’s immune status, the Immune Status Questionnaire (ISQ) was completed [25]. Current perceived immune fitness was assessed using a 1-item scale ranging from 0 (very poor) to 10 (excellent) [25,26]. The scale has a Cronbach’s alpha of 0.80 [25] and was included as previous research found an association between having hangovers and immune status [27,28]. Additionally, a short scale was used to assess baseline mood. The six items reflected the subscales of the short version of the Profiles of Mood States (POMS-SF) [29], and included tension/stress, anxiety, depression, being active, fatigue, and anger/hostility. The items were scored on a scale ranging from 0 (absent) to 10 (extreme). The 11-point scale has successfully been used in previous research [30,31], which showed that single item visual analog scales are just as sensitive and reliable as full-scale construct assessments of mood states like depression [32], fatigue [33], or quality of life [34].

Neuroticism was assessed with the neuroticism scale of the Eysenck Personality Questionnaire-Revised Short Scale (EPQ-RSS) [35,36]. The neuroticism scale consists of 12 items that can be answered with ‘yes’ or ‘no’, which correspond to the values of 1 and 0, respectively. The sum score of items ranges from 0 to 12, with higher scores implying more neuroticism. Cronbach’s alpha of the neuroticism scale is 0.82 [36].

In addition to demographics and baseline mood, various other assessments regarding alcohol consumption and mood were made for the past three days (referred to as ‘today’, ‘yesterday’, and ‘2 days ago’). For each of these days, subjects reported their alcohol consumption. Both the number of alcoholic drinks and the time frame of consumption were assessed. The estimated BAC was computed with a modified Widmark equation [37]. Subjective intoxication was rated on a scale ranging from 0 (sober) to 10 (very drunk) [38]. To assess the current mood while drinking, participants rated their mood state while drinking, including being ‘angry/hostile/irritable’ and being ‘depressed/sad’ on scales ranging from 0 (absent) to 10 (extreme). Total sleep time was assessed and subjects rated their sleep quality on a scale ranging from 0 to 10 [39,40]. Regarding next-day effects, hangover severity was scored with a 1-item severity score, ranging from 0 (absent) to 10 (extreme) [24]. Using the same 0–10 scale, ‘fatigue, sleepiness’, ‘stress’, and ‘guilt about drinking’ were also assessed as measures of current (hangover) mood.

Statistical analyses were conducted with SPSS (IBM SPSS Statistics for Windows, version 25.0, released in 2013; IBM Corp., Armonk, NY, USA). Mean and standard deviation (SD) were computed for each variable. Outlier data (alcohol intake on evening > +3SD of group average) were omitted from the analyses.

For each test day, participants were independently allocated to the ‘no hangover’ or ‘hangover’ group. This was based on the reported absence (score 0) or presence (score 1–10) of a hangover for that particular day. Thus, group sizes differed between the three days, and individual subjects could be allocated to the hangover group on one day, but to the no hangover group on another day, depending on the reported presence and absence of hangover for that particular day.

All statistical analyses were conducted separately for each of the three days. Most study outcome variables did not follow a normal distribution. Therefore, nonparametric statistics were used to analyze the data. To compare demographics and baseline mood between the hangover and the no hangover group, independent-samples Mann–Whitney U tests were used.

Spearman’s rho correlations were computed between drinking variables and mood outcomes. Results were considered significant if *p* < 0.05. Linear stepwise regression analyses (for which independent variables do not need to be normally distributed or continuous) were conducted to determine which variables (i.e., demographics, mood, and drinking variables) were significant predictors of (a) having a hangover (yes/no) and of (b) hangover severity (1–10 score on the single-item hangover severity assessment). Further, linear stepwise regression analyses were conducted to determine which of the assessed variables were significant predictors of (c) the amount of consumed alcohol and of (d) subjective intoxication on the evenings preceding the next-day alcohol hangover. Analyses were conducted for the whole sample, and for men and women separately.

## 3. Results

The survey was completed by *N* = 331 subjects. Their demographics and baseline mood are summarized in Table 1. A total of *N* = 143 subjects (43.2% of the sample) reported having a hangover on the day of the assessment (referred to as ‘today’ throughout the article). Table 1 contrasts their demographics and past day drinking behaviors with the *N* = 188 subjects who did not report having a hangover. The comparisons revealed that subjects with a hangover scored significantly higher on some of the baseline mood scales and perceived immune fitness compared to subjects who reported no hangover. However, it should be noted that the magnitudes of the observed differences were small (<1 on 11-point scales).

Variables related to alcohol consumption and mood (rated separately for mood during drinking and mood while hungover) are summarized in Table 2. For subjects with a hangover, the partial correlation with hangover severity (controlled for estimated BAC) is also indicated.

As can be seen in Table 2, the hangover group significantly differed from the no hangover group in almost all of the drinking-, sleep-, and current mood-associated variables. Significant partial correlations (controlling for estimated BAC) were found between hangover severity and subjective intoxication (being drunk) (r = 0.453, *p* < 0.0001), between hangover severity and total sleep time (r = −0.226, *p* = 0.009), and between hangover severity and sleep quality (r = −0.183, *p* = 0.036). There were no significant correlations between hangover severity and baseline mood or neuroticism. Ratings of mood while drinking did not significantly correlate with hangover severity. However, significant correlations were found between hangover severity and ‘fatigue, sleepiness’ experienced during hangover (r = 0.514, *p* < 0.0001), between hangover severity and ‘stress’ experienced during hangover (r = 0.423, *p* < 0.0001), and between hangover severity and ‘guilt about drinking’ experienced during hangover (r = 0.361, *p* < 0.0001). A similar pattern of outcomes was seen for the other two days that were assessed (Table 2).

Table 3 and Table 4 present the results of stepwise linear regression analyses including all the variables summarized in Table 1 and Table 2. Table 3 and Table 4 list those predictors that significantly contributed to each regression model (once while excluding and once while including “next-day” variables). The percentage that each particular variable contributed to the model (R^2^) and the beta coefficient (β) are also included.

The analysis of the current day revealed that three variables accounted for 45.2% of the variance in overall hangover severity. With regard to the variance explained by individual variables, subjective intoxication was the strongest predictor of hangover severity (43.0%), followed by baseline fatigue (1.5%) and sleep quality (0.7%). The addition of the ‘next day’ variables mood and guilt experienced while hungover yielded a model where four variables accounted for 56.1% of the variance in overall hangover severity. Subjective intoxication was again the strongest predictor of hangover severity (43.0%), followed by fatigue while hungover (8.7%), guilt about drinking while hungover (4.0%), and stress while hungover (0.4%). Regression analyses for the other two days of assessments yielded comparable results, as subjective intoxication was always the best predictor (Table 3 and Table 4). Table 3 and Table 4 also show the outcomes of separate regression analyses for men only and women only. These analyses again yielded comparable results as subjective intoxication was the most important predictor of hangover severity. In contrast to subjective intoxication, the amount of consumed alcohol and estimated BAC only had a marginal impact on hangover severity across all models. The demographic and mood variables that affected alcohol consumption are summarized in Table 5. For the ‘today’ data, a stepwise regression analysis revealed that three variables accounted for 25.0% of the variance in the amount of alcohol consumed (Table 5). The analysis showed that the number of smoked cigarettes was the strongest predictor and explained the most variance in the amount of consumed alcohol (16.4%). This was followed by sex (6.2%) and weekly alcohol consumption (2.4%). The data for the other two days yielded similar results, as the number of smoked cigarettes was always a relevant predictor of the amount of consumed alcohol.

The demographic and mood variables that affected alcohol consumption are summarized in Table 6. Three variables accounted for 41.0% of the variance in subjective intoxication (drunkenness) (Table 6). The analysis showed that the amount of consumed alcohol was the strongest predictor of subjective intoxication (37.9%), followed by feeling angry while drinking (2.1%) and age (1.0%). The data for the other two days (Table 6) yielded similar results: alcohol intake on the respective evening was always the most important predictor of drunkenness. Taken together, baseline mood and feeling more ‘angry/hostile’ or ‘depressed’ while drinking had only marginal effects on the amount of consumed alcohol (<5%). The overall variance of alcohol consumption explained across the models was low, with the number of cigarettes smoked being the most important predictor. This suggests that, instead of mood, other (not assessed) variables are more important predictors of the amount of alcohol consumption. The models for subjective intoxication were more robust, with the amount of consumed alcohol being the best predictor of subjective intoxication. Baseline mood and feeling angry/hostile or depressed while drinking had only small effects on subjective intoxication.

**Figure 1:**
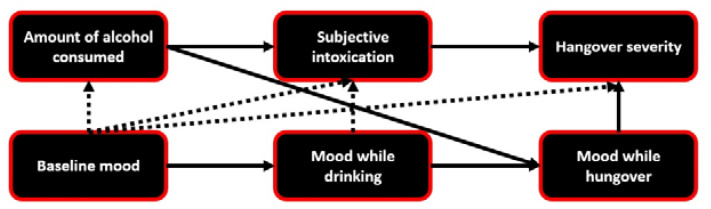
Associations between drinking variables, mood, and hangover severity. Lines represent significantly contributing variables to the regression analyses. Dashed lines connect variables that contributed less than 5% to the associations. The actual percentages are listed in Table 3, Table 4, Table 5 and Table 6.

A summary of all findings is presented in Figure 1.

## 4. Discussion

The current study aimed to verify and extend the observations by Harburg et al. in an international sample of young adults by applying a 1-item overall hangover severity scale to investigate whether baseline mood, mood while drinking, as well as mood during hangover, were associated with and/or predicted current and retrospective hangover severity. In contrast to previous reports [15,21,22], the findings of our study suggest that even though mood while drinking seemed to differ between hungover and non-hungover populations, this factor has a rather negligible impact on hangover severity. Instead, variables related to alcohol intake (in particular, subjective intoxication and estimated BAC) and sleep (in particular, sleep quality) were much more strongly related to hangover severity. Feeling stressed and fatigued during hangover were also significantly associated with hangover severity, confirming that mood changes accompany alcohol hangover. Finally, guilt was experienced most frequently by drinkers in the hangover group. Guilt about drinking significantly correlated with both the amount of alcohol consumed and with hangover severity.

The confirmatory regression analyses further supported our conclusions. The obtained models revealed that subjective intoxication (drunkenness) was the most important contributor to hangover severity. In comparison to that, mood while drinking had no relevant impact on hangover severity. Mood while hungover and guilt about drinking while hungover significantly contributed to the model predicting hangover severity, but it should also be noted that stress, fatigue, and guilt during hangover are most likely the consequences, rather than the cause of hangover severity.

Regression models predicting subjective intoxication revealed that the most important contributing factor was the amount of consumed alcohol. For both subjective intoxication and the amount of alcohol consumed, the regression models revealed that baseline mood and mood during drinking only had a small contribution to the models, if any (usually < 5%). At first sight, this might be regarded as comparable to the findings reported by Harburg et al. [22], who reported that being angry when high/drunk accounted for 3% (2%) of the observed hangover severity variance in men (women) and that being depressed when high/drunk accounted for another 1% of the variance in hangover severity observed for men when using composite HSI scores. Yet, our findings need to be interpreted within the context that out of all the assessed factors, the mood variables tended to explain the least variance, thus being the least suitable predictors of hangover severity.

The observation that subjective intoxication was the most important predictor of hangover severity is in line with results of previous studies [41,42,43]. One of these studies suggested that ‘consuming more alcohol than usual’ was an even better predictor of hangover severity than subjective intoxication [43]. Unfortunately, this variable was not included in the current study. We therefore recommend assessing how much alcohol is typically consumed at an average drinking occasion. This might be done either with individualized questions or with the help of (semi)structured clinical interview tools. Furthermore, it might also be beneficial to include measures of overall alcohol sensitivity, such as the Self-Rating of the Effects of Alcohol (SRE) form [44] or the Alcohol Sensitivity Questionnaire (ASQ) [45]. Another potential limitation of the current study was the relatively young sample, which makes it unclear to what extent the results can be generalized to other age groups. We therefore recommend assessing samples that cover wide age ranges, whenever possible. The data also relied on retrospective self-reports, which might have suffered from recall bias in some participants and which might potentially have led to the smaller number of hangovers reported with increasing recall period. Therefore, retrospective hangover assessments should ideally not be averaged over days that differ in recall period. Retrospective assessments also mean that BAC was not assessed while drinking, but instead calculated using the Widmark formula [37]. Given the possibility of recall bias and individual differences, the BAC was therefore reported as an estimate throughout this article. If possible, the BAC should ideally be determined on the night of drinking, but it should also be kept in mind that the measurement itself and the presence of investigators might induce bias, thus making drinking behavior less naturalistic [46].

Research on the relationship between smoking and the presence and severity of hangover is limited, and this is an important topic for future research. Table 3 and Table 4 show that both cigarette smoking and drug use were not significant predictors of hangover severity or subjective intoxication. However, this observation is in contrast to previous research that found smoking to significantly increase the odds of hangover incidence and hangover severity [47]. Our analysis did reveal that the number of cigarettes smoked was the strongest predictor of the amount of alcohol consumed. This observation is in line with other research showing that drinking and smoking often go hand in hand [48].

Finally, we examined possible sex differences in variables contributing to hangover severity. Previous research showed that the presence and severity of hangover symptoms did not relevantly differ between men and women at comparable BAC levels [49,50]. In the current study, conducting the statistical analysis separately for men and women revealed that men consumed significantly more alcohol than women, but we found no important sex differences in which variables significantly contributed to hangover severity. Across all analyses, subjective intoxication was the most important predictor of hangover severity. While mood during drinking had no relevant impact, mood during hangover was clearly associated with hangover severity.

## Figures and Tables

**Table 1:** Demographics and baseline mood.

	Total Sample	Hangover	No Hangover
**Demographics**			
*N* (%)	331 (100.0%)	143 (43.2%)	188 (56.8%)
Age (years)	23.6 (4.2)	23.5 (4.3)	23.6 (4.1)
Sex (m/f)	143/188	81/62	63/125
BMI (kg/m^2^)	23.5 (3.9)	23.8 (4.4)	23.2 (3.5)
Usual weekly alcohol consumption (units)	11.5 (11.0)	13.3 (12.1) *	10.2 (9.8)
Past year’s immune fitness (ISQ)	7.0 (2.3)	6.9 (2.2)	7.1 (2.3)
Perceived immune fitness	8.0 (1.6)	7.8 (1.6) *	8.1 (1.5)
**Baseline Mood Ratings**			
Tension, stress	1.8 (1.8)	1.9 (1.8)	1.7 (1.7)
Anxiety	1.5 (1.9)	1.5 (1.9)	1.5 (1.9)
Depression	0.7 (1.5)	0.8 (1.7) *	0.6 (1.3)
Being active	5.4 (2.8)	5.8 (2.6) *	5.1 (2.9)
Fatigue	3.5 (2.6)	4.1 (2.6) *	3.1 (2.6)
Anger, hostility	0.9 (1.6)	1.1 (1.8) *	0.7 (1.4)
Neuroticism	2.1 (2.2)	2.2 (2.2)	2.0 (2.2)

Mean and standard deviation (between brackets) are shown. Significant differences (*p* < 0.05) between the hangover and the no hangover group are indicated by *. No significant partial correlations with hangover severity were found (*p* < 0.05), controlling for estimated BAC. Abbreviations: BMI = body mass index, ISQ = Immune Status Questionnaire, BAC = blood alcohol concentration.

**Table 2:** Study outcomes and their association with hangover severity.

	Today		Yesterday		Two Days Ago	
	Hangover	No Hangover	Hangover	No Hangover	Hangover	No Hangover
*n* (%)	143	188	122	208	87	243
**Drinking variables**						
Alcohol consumed (units)	12.3 (7.3) *	4.0 (5.3)	11.4 (7.3) * ^†^	3.2 (4.6)	9.9 (7.4) * ^†^	1.9 (3.6)
Time spent drinking (h)	6.9 (3.5) *	3.0 (3.8)	6.6 (4.1) * ^†^	4.3 (4.5)	6.5 (4.6) * ^†^	3.9 (4.1)
Estimated BAC (%)	0.16 (0.1) * ^†^	0.08 (0.1)	0.17 (0.2) * ^†^	0.06 (0.1)	0.14 (0.1) *^†^	0.05 (0.1)
Subjective intoxication	6.2 (2.5) * ^†^	1.7 (2.4)	6.1 (2.7) * ^†^	1.7 (2.3)	5.6 (3.1) * ^†^	1.0 (2.1)
Cigarettes smoked	3.7 (6.4) *	1.4 (3.8)	2.8 (5.4) *	1.2 (3.1)	2.8 (5.3) *	1.1 (3.0)
**Mood during drinking**						
Angry, hostile, irritable	0.7 (1.6) *	0.4 (1.2)	0.9 (2.0) * ^†^	0.4 (1.3)	0.6 (1.2) *	0.3 (1.1)
Depressed, sad	0.6 (1.5) *	0.3 (1.2)	0.9 (2.0) *	0.4 (1.3)	0.9 (1.7) *	0.5 (1.7)
**Sleep**						
Total sleep time (h)	6.2 (2.0) ^†^	7.3 (2.1)	6.5 (1.8) * ^†^	7.2 (1.9)	6.6 (1.9) * ^†^	6.9 (2.5)
Sleep quality	6.0 (2.5) * ^†^	6.7 (2.3)	6.7 (2.3) ^†^	6.7 (2.3)	6.4 (2.5) ^†^	6.2 (2.7)
**Next-day mood**						
Hangover severity	3.5 (2.5) *	0.0 (0.0)	3.7 (2.7) *	0.0 (0.0)	3.2 (2.3) *	0.0 (0.0)
Fatigue, sleepiness	4.7 (2.9) * ^†^	1.7 (2.4)	5.1 (2.6) * ^†^	1.5 (2.3)	4.5 (3.0) * ^†^	1.7 (2.6)
Stress	1.3 (2.1) * ^†^	0.4 (1.3)	1.7 (2.5) * ^†^	0.4 (1.2)	1.8 (2.6) * ^†^	0.3 (1.0)
Guilt about drinking	1.4 (2.3) * ^†^	0.2 (0.9)	1.4 (2.3) * ^†^	0.1 (0.6)	1.5 (2.4) * ^†^	0.1 (0.7)

Significant differences (*p* < 0.05) between the hangover and the no hangover group are indicated by *. Significant partial correlations (*p* < 0.05), controlling for estimated BAC, with hangover severity are indicated by ^†^. Abbreviation: BAC = blood alcohol concentration.

**Table 3:** Significant predictors of hangover severity (excluding next-day variables).

**Full Sample**	**Today (*n* = 313)**	**Yesterday (*n* = 243)**	**Two days ago (*n* = 175)**
	**Model: R^2^ = 45.2%**	**Model: R^2^ = 42.8%**	**Model: R^2^ = 43.1%**
Contributing variables	Subjective intoxication (R^2^ = 43.0%) (β = 0.644, *p* < 0.0001)	Subjective intoxication (R^2^ = 38.9%) (β = 0.524, *p* < 0.0001)	Subjective intoxication (R^2^ = 37.0%) (β = 0.561, *p* < 0.0001)
	Baseline fatigue (R^2^ = 1.5%) (β = 0.117, *p* = 0.006)	ISQ (R^2^ = 2.1%) (β = −0.165, *p* = 0.001)	Estimated BAC (R^2^ = 3.9%) (β = 0.420, *p* < 0.0001)
	Sleep quality (R^2^ = 0.7%) (β = −0.096, *p* = 0.024)	Estimated BAC (R^2^ = 1.8%) (β = 0.167, *p* = 0.004)	Alcohol intake evening (R^2^ = 1.2%) (β = −0.288, *p* = 0.022)
			Sleep quality (R^2^ = 1.0%) (β = −0.115, *p* = 0.049)
**Men only**	**Today (*n* = 120)**	**Yesterday (*n* = 110)**	**Two days ago (*n* = 91)**
	**Model: R^2^ = 36.2%**	**Model: R^2^ = 60.5%**	**Model: R^2^ = 44.3%**
Contributing variables	Subjective intoxication (R^2^ = 33.5%) (β = 0.570, *p* < 0.0001)	Subjective intoxication (R^2^ = 38.9%) (β = 0.485, *p* < 0.0001)	Subjective intoxication (R^2^ = 41.0%) (β = 0.818, *p* < 0.0001)
	Baseline anger, hostility (R^2^ = 2.7%) (β = 0.181, *p* = 0.015)	Stress while hungover (R^2^ = 12.3%) (β = 0.293, *p* < 0.0001)	Drinking time (R^2^ = 3.3%) (β = −0.263, *p* = 0.013)
		Fatigue while hungover (R^2^ = 3.6%) (β = 0.179, *p* = 0.019)	
		Estimated BAC (R^2^ = 3.1%) (β = 0.495, *p* = 0.002)	
		Weekly alcohol intake (R^2^ = 1.2%) (β = 0.159, *p* = 0.019)	
		Alcohol intake evening (R^2^ = 1.4%) (β = −0.404, *p* = 0.030)	
**Women only**	**Today (*n* = 133)**	**Yesterday (*n* = 132)**	**Two days ago (*n* = 83)**
	**Model: R^2^ = 41.7%**	**Model: R^2^ = 46.2%**	**Model: R^2^ = 44.0%**
Contributing variables	Subjective intoxication (R^2^ = 38.9%) (β = 0.580, *p* < 0.0001)	Subjective intoxication (R^2^ = 38.9%) (β = 0.560, *p* < 0.0001)	Subjective intoxication (R^2^ = 32.5%) (β = 0.392, *p* = 0.001)
	Total sleep time (R^2^ = 2.8%) (β = −0.183, *p* = 0.009)	Weekly alcohol intake (R^2^ = 3.2%) (β = −0.221, *p* = 0.001)	Current immune fitness (R^2^ = 9.1%) (β = −0.279, *p* = 0.001)
		ISQ (R^2^ = 2.4%) (β = −0.172, *p* = 0.010)	Estimated BAC (R^2^ = 2.1%) (β = 0.235, *p* = 0.036)
		Estimated BAC (R^2^ = 1.7%) (β = 0.207, *p* = 0.008)	

Linear stepwise regression analyses were conducted on the data of participants who reported having a hangover. The included variables were demographics, baseline mood, neuroticism, alcohol consumption variables, and sleep outcomes. The percentage of variance explained (adjusted R^2^), the unadjusted beta coefficient (β), and standard error (SE) are provided. Abbreviation: BAC = estimated blood alcohol concentration, ISQ = Immune Status Questionnaire.

**Table 4:** Significant predictors of hangover severity (including next-day variables).

**Full sample**	**Today (*n* = 313)**	**Yesterday (*n* = 243)**	**Two days ago (*n* = 175)**
	**Model: R^2^ = 56.1%**	**Model: R^2^ = 58.4%**	**Model: R^2^ = 56.7%**
Contributing variables	Subjective intoxication (R^2^ = 43.0%) (β = 0.472, *p* < 0.0001)	Subjective intoxication (R^2^ = 38.9%) (β = 0.331, *p* < 0.0001)	Subjective intoxication (R^2^ = 37.0%) (β = 0.319, *p* < 0.0001)
	Fatigue while hungover (R^2^ = 8.7%) (β = 0.237, *p* < 0.0001)	Stress while hungover (R^2^ = 13.0%) (β = 0.220, *p* < 0.0001)	Stress while hungover (R^2^ = 11.8%) (β = 0.351, *p* = 0.0001)
	Guilt about drinking (R^2^ = 4%) (β = 0.186, *p* < 0.0001)	Fatigue while hungover (R^2^ = 3.9%) (β = 0.227, *p* < 0.0001)	Estimated BAC (R^2^ = 4.5%) (β = 0.227, *p* < 0.0001)
	Stress while hungover (R^2^ = 0.4%) (β = 0.093, *p* = 0.048)	Guilt about drinking (R^2^ = 2.0%) (β = 0.171, *p* = 0.001)	Fatigue while hungover (R^2^ = 2.2%) (β = 0.188, *p* = 0.001)
		Estimated BAC (R^2^ = 0.6%) (β = 0.107, *p* = 0.028)	Angry while drinking (R^2^ = 1.6%) (β = −0.142, *p* = 0.006)
**Men only**	**Today (*n* = 120)**	**Yesterday (*n* = 110)**	**Two days ago (*n* = 91)**
	**Model: R^2^ = 50.3%**	**Model: R^2^ = 50.8%**	**Model: R^2^ = 66.5%**
Contributing variables	Subjective intoxication (R^2^ = 33.5%) (β = 0.423, *p* < 0.0001)	Subjective intoxication (R^2^ = 38.9%) (β = 0.578, *p* < 0.0001)	Subjective intoxication (R^2^ = 41.0%) (β = 0.618, *p* < 0.0001)
	Fatigue while hungover (R^2^ = 12.5%) (β = 0.306, *p* < 0.0001)	Estimated BAC (R^2^ = 4.8%) (β = 0.657, *p* < 0.0001)	Stress while hungover (R^2^ = 17.1%) (β = 0.426, *p* < 0.0001)
	Guilt about drinking (R^2^ = 4.3%) (β = 0.234, *p* = 0.001)	Baseline anger, hostility (R^2^ = 3.2%) (β = 0.226, *p* = 0.001)	Estimated BAC (R^2^ = 4.1%) (β = 0.522, *p* < 0.0001)
		Alcohol intake evening (R^2^ = 2.1%) (β = −0.551, *p* = 0.008)	Alcohol intake evening (R^2^ = 2.1%) (β = −0.437, *p* = 0.009)
		Weekly alcohol intake (R^2^ = 1.8%) (β = 0.155, *p* = 0.033)	Angry while drinking (R^2^ = 1.7%) (β = −0.143, *p* = 0.024)
**Women only**	**Today (*n* = 133)**	**Yesterday (*n* = 132)**	**Two days ago (*n* = 83)**
	**Model: R^2^ = 54.4%**	**Model: R^2^ = 61.4%**	**Model: R^2^ = 53.1%**
Contributing variables	Subjective intoxication (R^2^ = 38.9%) (β = 0.419, *p* < 0.0001)	Fatigue while drinking (R^2^ = 39.6%) (β = 0.226, *p* = 0.002)	Subjective intoxication (R^2^ = 32.5%) (β = 0.358, p < 0.0001)
	Fatigue while hungover (R^2^ = 9.8%) (β = 0.322, *p* < 0.0001)	Guilt about drinking (R^2^ = 12.4%) (β = 0.250, *p* < 0.0001)	Fatigue while hungover (R^2^ = 9.1%) (β = 0.325, *p* < 0.0001)
	Guilt about drinking (R^2^ = 4.1%) (β = 0.239, *p* < 0.0001)	Subjective intoxication (R^2^ = 5.5%) (β = 0.343, *p* < 0.0001)	Stress while hungover (R^2^ = 5.9%) (β = 0.224, *p* = 0.012)
	Baseline anxiety (R^2^ = 1.6%) (β = −0.139, *p* = 0.021)	Stress while drinking (R^2^ = 2.3%) (β = 0.196, *p* = 0.005)	Baseline fatigue (R^2^ = 3.3%) (β = −0.220, *p* = 0.006)
		Weekly alcohol intake (R^2^ = 1.8%) (β = −0.147, *p* = 0.010)	Current immune fitness (R^2^ = 2.3%) (β = −0.189, *p* = 0.031)

Linear stepwise regression analyses were conducted on the data of participants who reported having a hangover. The included variables were demographics, baseline mood, neuroticism, alcohol consumption variables, sleep outcomes, and next-day variables on mood while hungover, as well as guilt about drinking. The percentage of variance explained (adjusted R^2^), the standardized beta coefficient (β), and *p*-value are given.

**Table 5:** Significant predictors of alcohol consumption.

**Full sample**	**Today (*n* = 318)**	**Yesterday (*n* = 315)**	**Two days ago (*n* = 317)**
	**Model: R^2^ = 25.0%**	**Model: R^2^ = 20.7%**	**Model: R^2^ = 26.1%**
Contributing variables	Cigarettes smoked (R^2^ = 16.4%) (β = 0.320, *p* < 0.0001)	Cigarettes smoked (R^2^ = 14.0%) (β = 0.293, *p* < 0.0001)	Cigarettes smoked (R^2^ = 16.6%) (β = 0.353, *p* < 0.0001)
	Sex (R^2^ = 6.2%) (β = −0.237, *p* < 0.0001)	Weekly alcohol intake (R^2^ = 6.7%) (β = 0.275, *p* < 0.0001)	Weekly alcohol intake (R^2^ = 3.6%) (β = 0.165, *p* = 0.001)
	Weekly alcohol intake (R^2^ = 2.4%) (β = 0.167, *p* = 0.001)		Angry while drinking (R^2^ = 3.1%) (β = 0.194, *p* < 0.0001)
			Baseline anger, hostility (R^2^ = 1.6%) (β = −0.148, *p* = 0.003)
			Sex (R^2^ = 1.4%) (β = −0.132, *p* = 0.009)
**Men only**	**Today (*n* = 140)**	**Yesterday (*n* = 139)**	**Two days ago (*n* = 140)**
	**Model: R^2^ = 12.1%**	**Model: R^2^ = 23.3%**	**Model: R^2^ = 22.2%**
Contributing variables	Cigarettes smoked (R^2^ = 8.8%) (β = 0.361, *p* < 0.0001)	Cigarettes smoked (R^2^ = 17.1%) (β = 0.318, *p* < 0.0001)	Cigarettes smoked (R^2^ = 12.9%) (β = 0.384, *p* < 0.0001)
	Baseline anxiety (R^2^ = 3.3%) (β = −0.204, *p* = 0.014)	Weekly alcohol intake (R^2^ = 6.2%) (β = 0.279, *p* = 0.001)	Angry while drinking (R^2^ = 6.9%) (β = 0.304, *p* < 0.0001)
			Baseline anger, hostility (R^2^ = 2.4%) (β = −0.175, *p* = 0.025)
**Women only**	**Today (*n* = 177)**	**Yesterday (*n* = 175)**	**Two days ago (*n* = 176)**
	**Model: R^2^ = 24.6%**	**Model: R^2^ = 12.3%**	**Model: R^2^ = 22.4%**
Contributing variables	Cigarettes smoked (R^2^ = 22.1%) (β = 0.442, *p* < 0.0001)	Weekly alcohol intake (R^2^ = 8.0%) (β = 0.261, *p* < 0.0001)	Cigarettes smoked (R^2^ = 16.6%) (β = 0.369, *p* < 0.0001)
	Weekly alcohol intake (R^2^ = 2.5%) (β = 0.174, *p* = 0.010)	Cigarettes smoked (R^2^ = 4.3%) (β = 0.219, *p* = 0.003)	Weekly alcohol intake (R^2^ = 5.8%) (β = 0.253, *p* < 0.0001)

Linear stepwise regression analyses were conducted on the data of participants who reported having a hangover. The included variables were demographics, baseline mood, neuroticism, and mood while drinking. The percentage of variance explained (adjusted R^2^), the standardized beta coefficient (β), and *p*-value are given.

**Table 6:** Significant predictors of subjective intoxication.

**Full sample**	**Today (*n* = 254)**	**Yesterday (*n* = 244)**	**Two days ago (*n* = 175)**
	**Model: R^2^ = 41.0%**	**Model: R^2^ = 45.5%**	**Model: R^2^ = 54.5%**
Contributing variables	Alcohol intake evening (R^2^ = 37.9%) (β = 0.617, *p* < 0.0001)	Alcohol intake evening (R^2^ = 39.8%) (β = 0.623, *p* < 0.0001)	Alcohol intake evening (R^2^ = 52.2%) (β = 0.735, *p* < 0.0001)
	Angry while drinking (R^2^ = 2.1%) (β = 0.144, *p* = 0.003)	Angry while drinking (R^2^ = 3.8%) (β = 0.196, *p* < 0.0001)	Baseline fatigue (R^2^ = 2.3%) (β = 0.160, *p* = 0.002)
	Age (R^2^ = 1.0%) (β = −0.112, *p* = 0.021)	Age (R^2^ = 1.9%) (β = −0.145, p = 0.002)	
**Men only**	**Today (*n* = 120)**	**Yesterday (*n* = 110)**	**Two days ago (*n* = 91)**
	**Model: R^2^ = 49.3%**	**Model: R^2^ = 58.4%**	**Model: R^2^ = 67.8%**
Contributing variables	Alcohol intake evening (R^2^ = 44.3%) (β = 1.060, *p* < 0.0001)	Alcohol intake evening (R^2^ = 48.4%) (β = 1.033, *p* < 0.0001)	Alcohol intake evening (R^2^ = 55.1%) (β = 1.113, *p* < 0.0001)
	Estimated BAC (R^2^ = 3.1%) (β = −0.438, *p* = 0.006)	Angry while drinking (R^2^ = 8.0%) (β = 0.252, *p* < 0.0001)	Estimated BAC (R^2^ = 5.7%) (β = −0.467, *p* < 0.0001)
	Baseline being active (R^2^ = 1.9%) (β = 0.151, *p* = 0.022)	Estimated BAC (R^2^ = 1.8%) (β = −0.372, *p* = 0.018)	Baseline being active (R^2^ = 3.6%) (β = 0.148, *p* = 0.019)
			Baseline neuroticism (R^2^ = 1.9%) (β = 0.177, *p* = 0.006)
			Age (R^2^ = 1.5%) (β = −0.133, *p* = 0.031)
**Women only**	**Today (*n* = 133)**	**Yesterday (*n* = 133)**	**Two days ago (*n* = 83)**
	**Model: R^2^ = 33.0%**	**Model: R^2^ = 44.3%**	**Model: R^2^ = 49.5%**
Contributing variables	Alcohol intake evening (R^2^ = 27.8%) (β = 0.522, *p* < 0.0001)	Alcohol intake evening (R^2^ = 34.7%) (β = 0.908, *p* < 0.0001)	Alcohol intake evening (R^2^ = 49.5%) (β = 0.708, *p* < 0.0001)
	Angry while drinking (R^2^ = 3.5%) (β = 0.197, *p* = 0.006)	Age (R^2^ = 4.7%) (β = −0.222, *p* = 0.001)	
	Age (R^2^ = 1.7%) (β = −0.147, *p* = 0.040)	Estimated BAC (R^2^ = 2.7%) (β = −0.386, *p* = 0.006)	
		Depressed while drinking (R^2^ = 2.2%) (β = 0.160, *p* = 0.016)	

Linear stepwise regression analyses were conducted on the data of participants who reported having a hangover. The included variables were demographics, baseline mood, neuroticism, mood while drinking, and amount of alcohol consumed (alcohol intake during the evening). The percentage variance explained (adjusted R^2^), the standardized beta coefficient (β), and *p*-value are given.
